# Assessing Physical Activity and Perceived Barriers Among Physicians in Primary Healthcare in Makkah City, Saudi Arabia

**DOI:** 10.7759/cureus.23605

**Published:** 2022-03-29

**Authors:** Dania M Melebari, Adeel Ahmed Khan

**Affiliations:** 1 Preventive Medicine, Saudi Board of Preventive Medicine, Ministry of Health, Makkah, SAU; 2 Epidemiology and Public Health, Saudi Board of Preventive Medicine, Ministry of Health, Makkah, SAU

**Keywords:** saudi arabia, makkah, primary health care centers, barriers, physical activity

## Abstract

Introduction

Advocating a healthy lifestyle is the cornerstone of primary healthcare physicians. As physicians are the ultimate role models for patients regarding health and well-being, we focused on physicians working in primary healthcare centers (PHCs) in Makkah because they work on the frontline of disease prevention and are considered the first point of contact for patients entering the health system. This study aimed to estimate the physical activity levels in physicians working in the PHCs of Makkah and any perceived barriers to engage in physical activity.

Methodology

We conducted a cross-sectional study at PHCs in Makkah from October 2021 to December 2021. We used a multistage cluster random sampling technique to select primary healthcare physicians in Makkah city. We recruited 196 physicians working in PHCs for this study. We used the short version of the International Physical Activity Questionnaire to measure physical activity levels, and we used the barriers to being active questionnaire to identify the barriers. Descriptive analysis was performed using frequencies. Bivariate associations between the most frequently reported barriers and sociodemographic variables were determined using the chi-square test, Student t-test, and analysis of variance via IBM SPSS Statistics for Windows, version 22.0 (Armonk, NY: IBM Corp.).

Results

Approximately 45.9% of physicians were overweight, while 69.4% were not gym members. In the seven days prior to answering the surveys, approximately 71.9% did not report any vigorous activity, and 30.6% had performed moderate activity. The most significant barrier to being active was a lack of time (70.9%), followed by a lack of resources (69.9%). In bivariate analysis, we noted a significant negative relationship between gym membership, vigorous and moderate physical activity, and perceived barriers scores (p<0.001).

Conclusion

Most of the physicians in PHCs are not physically active. The main barrier to their physical activity is lack of time. There is a need to encourage them and motivate them to be physically active to model more healthy behaviors to the general population.

## Introduction

Physical activity is defined as any bodily movement produced by skeletal muscles that expend energy, including working, playing, running, home chores, traveling, and recreational pursuits; regular physical activity is an essential aspect of good health [1]. Globally, a lack of physical activity was the fourth leading cause of death (6%) [2,3]. The World Health Organization (WHO) recommends that adults aged 18-64 years should "do at least 150 minutes of moderate-intensity aerobic physical activity weekly or at least 75 minutes of vigorous-intensity aerobic physical activity weekly or an equivalent combination of moderate and vigorous-intensity activity" to improve health and decrease cardio-respiratory and muscular diseases and reduce the risk of noncommunicable diseases (NCDs) and depression [4,5]. The WHO estimated that more than 30% of the world population and 30-70% of people in Eastern Mediterranean (EMRO) countries do not achieve the minimum recommended physical activity level [6,7].

Over the past few decades, Saudi Arabia has become a more developed and westernized country than in previous years. With these changes came an increased prevalence of overweight and obesity, even in young people. The WHO estimates the prevalence of physical inactivity among Saudi children, youth, and adults as 57%, 71%, and 80%, respectively [8]. This trend puts the population at risk for increased rates of NCDs-related mortality. Competitor cultures bear some responsibility because the combination of continuous ancient Saudi cultural practices, economic prosperity, inactivity, and lack of physical activity has created an obesogenic setting that promotes unhealthy, inactive lifestyles and weight gain. The WHO stresses that physical activity promotion ought to be a vital public health objective [8].

High body weight and obesity are global concerns, with more than one in five individuals qualifying as overweight worldwide [9]. These conditions are linked with excessive rates of NCDs associated with type 2 diabetes mellitus, hypertension, and cardiovascular diseases [10]. Although most Saudi people are aware of the benefits of regular physical activity, many of them experience barriers to daily exercise, especially healthcare workers. These barriers can be internal or personal causes, such as not liking physical activity, not seeing its usefulness, feeling lazy, or thinking that they are not competent to practice such activities. They also experience external barriers, such as lack of time, resources, social support, and stressful work or energy loss [11].

There is a strong association between physical activity and psychological benefits, leading to increased quality of work among physicians. Exploring physicians' barriers to physical activity can help improve their quality of life [12]. When counseling patients, physicians report difﬁculties in recommending physical activities that the physicians themselves cannot engage in due to internal and external barriers [13]. Like their patients, physicians experience long working hours with prolonged periods of inactivity. Physicians in primary healthcare centers (PHCs) should be motivated to move and reduce their risk of inactivity-related issues. Due to a lack of evidence regarding the level of physical activity among physicians working in PHCs in Makkah, we conducted this study to assess their physical activity levels and investigate the perceived barriers to exercise.

## Materials and methods

We conducted this cross-sectional study from October 2021 to December 2021. The study population included all physicians serving at PHCs affiliated with the Ministry of Health (MOH) in Makkah. Physicians on vacation were excluded from the study. We used multistage cluster random sampling to divide all 43 PHCs in Makkah into seven sectors according to their locations. Each sector contained six to seven centers. Then, we choose three to four primary healthcare centers randomly from every sector. A total of 20 PHCs were included in our study. Due to the coronavirus disease 2019 pandemic, the data were collected manually from each physician in the PHCs via iPad (Apple Inc.: Cupertino, CA). Also, the electronic questionnaire was distributed through WhatsApp to the physicians who were busy in clinics during the time of data collection. Approximately 500 physicians worked in PHCs in Makkah during the study period. The overall response rate was 28%. We calculate the sample size by using OpenEpi version 3.01 (Seattle, WA: The Bill & Melinda Gates Foundation) according to the following criteria: confidence level of 95%, a margin of error of 5%, and an expected proportion of the population with adequate knowledge of 74.6% based on a similar study; the total sample size calculated was 185 physicians [2].

All necessary official approvals were fulfilled before start of data collection from the Institutional Review Board in Makkah with number (H-02-K-076-0320-274). An informed consent was taken and was given on the first page of the electronic questionnaire. Collected data were dealt with confidentially.

We collected the demographic data on participants gender, age, height, weight, body mass index (BMI), nationality, social status, specialty, work experience, income, history of chronic diseases, history of smoking or substance use, and gym membership; these were independent variables in our study. In addition to socio-economic and demographic data, we used two questionnaires to collect the data: the International Physical Activity Questionnaire (IPAQ) [10] to measure the level of physical activity and the Barriers to Being Active Quiz (BBAQ) from the Centers for Disease Control and Prevention (CDC) [14]. All data were coded according to the guidance in the IPAQ analysis tool.

The IPAQ short form is an instrument designed primarily for population surveillance of physical activity among adults (i.e., those aged 15 to 69 years). The reliability and validity of the questionnaire were tested across 12 countries (14 sites) in the year 2000. The findings suggested that it is suitable for studies of participation in physical activity [4,15]. The short form of IPAQ has seven items providing information on time spent walking, vigorous and moderate-intensity physical activities, and sedentary activity over the previous seven days. The IPAQ scores were categorized as vigorous, moderate, low, or no activity. Vigorous physical activities are defined as those “producing vigorous increases in respiration rate, heart rate, and sweating for at least 10 minutes” (>6 metabolic equivalents {ME}). IPAQ defines moderate physical activities as those that produce a moderate increase in respiration rate, heart rate, and sweating for at least 10 minutes (3 to 6 MET). IPAQ defines low activity as those that use <3 METs. One MET is the amount of oxygen consumed while sitting at rest with no activity [16]. Before participants had answered the questions, they were asked to think about any vigorous or moderate activities performed in the previous seven days during work, transportation, household activities, yard/garden activities, and leisure/sports activities [17,18].

The BBAQ is validated in terms of reproducibility and used internationally. It is a 21-item scale that measures seven self-reported barriers to being physically active. Every question has four choices consisting of very likely (score of three), somewhat likely (score of two), somewhat unlikely (score of one), and very unlikely (score of zero). The seven main barriers were social influence; fear of injury; and lack of time, energy, willpower, skill, and resources [19]. After calculating the score, we determined the main physical activity barrier for every participant.

Data entry and analysis were done using IBM SPSS Statistics for Windows, version 22.0 (Armonk, NY: IBM Corp.). Descriptive statistics were calculated as frequencies and percentages for qualitative variables and mean and standard deviations for quantitative variables. Bivariate associations between the most frequently reported barriers and sociodemographic variables were determined using the chi-square test, Student t-test, and analysis of variance. Statistical significance was defined as p<0.05.

## Results

Table 1 presents demographic data on the study population. Of 196 participants, 126 were female (64.3%) and 70 were male (35.7%). Most participants were Saudi physicians (n=176, 89.8%), with only 20 participants who were non-Saudi (10.2%) physicians. The mean age of participants was 34.1 ± 6.5 years (range: 24 to 59 years), and most participants were married (n=143, 73%). The study included family medicine consultants (16.8%), family medicine specialists (22.4%), family medicine residents (22.4%), general physicians (27%), and physicians from other specialties who had rotations at PHCs (8.7%). About 27.6% of the physicians in the study have four to seven years of professional experience, and almost as many as 27% have more than 10 years of experience. The majority earned more than 20,000 Saudi riyals (about 5332 USD) monthly (44.4%). Most physicians reported no chronic diseases (85.2%), and most respondents never smoke (65.3%).

**Table 1:** Demographic characteristics of the study population (n=196). BMI: body mass index; SD: standard deviation; SR: Saudi riyal

Demographic data		n	%
Age in years	<30	44	22.4
	30-40	122	62.2
	41-50	21	10.7
	>50	9	4.6
Mean ± SD		34.1 ± 6.5	
Gender	Male	70	35.7
	Female	126	64.3
Nationality	Saudi	176	89.8
	Non-Saudi	20	10.2
Marital status	Single	46	23.5
	Married	143	73.0
	Divorced and widowed	7	3.6
BMI (kg/m^2^)	Underweight (<18.5)	6	3.1
	Normal weight (18.5-24.9)	70	35.7
	Overweight (25-29.9)	90	45.9
	Obese (≥30)	30	15.3
Mean ± SD		26.489 ± 4.928	
Specialty	General physician	53	27.0
	Family medicine resident	44	22.4
	Family medicine specialist	49	25.0
	Family medicine consultant	33	16.8
	Others	17	8.7
Work experience in years	1-4	49	25.0
	4-7	54	27.6
	7-10	40	20.4
	>10	53	27.0
Monthly income in SR	<10,000	9	4.6
	10,000-14,999	39	19.9
	15,000-20,000	61	31.1
	>20,000	87	44.4
Other business	Yes	12	6.1
	No	184	93.9
Smoking	Regularly	18	9.2
	Occasionally	36	18.4
	Ex-smoker	14	7.1
	Never smoked	128	65.3
Any chronic disease	Yes	29	14.8
	No	167	85.2

Participants’ mean BMI was 26.4 kg/m^2^ (range: 14.5 to 54.1 kg/m^2^). Ninety participants were overweight (45.9%). More than half of the sample (69.4%) did not have a gym membership. Most participants (71.9%) reported not engaging in vigorous physical activity over the past seven days (like digging, aerobics, heavy lifting, or fast bicycling). Only 28.1% reported engaging in vigorous activity an average of 3.1 ± 1.4 days per week for an average of 39.3 ± 24.1 minutes per session.

Regarding moderate physical activity like doubles tennis, carrying light loads, or bicycling regularly, 30.6% of respondents reported doing moderate exercises 3.1 ± 1.3 days per week for 29.7 ± 15.0 minutes per day. However, 69.4% of participants did not perform any moderate activity. Most of the participants (69.9%) spent an average of 38.5 ± 68.5 minutes walking for 4.3 ± 2.2 days weekly. The participants' sitting duration ranged from one to 18 hours daily for a mean of 8.6 ± 3.1 hours (Table 2).

**Table 2:** Level of physical activity among study population (n=196). SD: standard deviation

Physical activity		n	%
Gym membership	Yes	60	30.6
	No	136	69.4
Vigorous physical activity	Yes	55	28.1
	No	141	71.9
Duration	Mean ± SD (days)	3.1 ± 1.4	
	Mean ± SD (minutes)	39.3 ± 24.1	
Moderate physical activity	Yes	60	30.6
	No	136	69.4
Duration	Mean ± SD (days)	3.1 ± 1.3	
	Mean ± SD (minutes)	29.7 ± 15.0	
Walking	Yes	137	69.9
	No	59	30.1
Duration	Mean ± SD (days)	4.3 ± 2.2	
	Mean ± SD (minutes)	38.5 ± 68.5	
Duration of sitting	Mean ± SD (hours)	8.6 ± 3.1	

In Table 3, barriers to physical activity were classified as weak and high after calculating the scores. Scores of ≥5 were considered high barriers. Lack of time for physical activity was a high barrier for most respondents (70.9%). Social influence was a weak barrier for most respondents (68.9%). Many respondents (63.8%) noted that lack of energy was a high barrier to physical activity. Most participants indicated a lack of willpower as a high barrier (53.1%). Fear of injury was a weak barrier among 91.3% of respondents. Lack of skills was also a weak barrier for most (83.7%). Lack of resources was a high barrier for 69.9% of respondents.

**Table 3:** Distribution of the characteristics of the study participants in relation to barriers towards practice of physical activity among physicians in PHCs of Makkah city (n=196). PHC: primary healthcare center; SD: standard deviation

Barrier	Barrier score				Scoring
	Weak		High		
	n	%	n	%	Mean ± SD
Lack of time	57	29.1%	139	70.9%	5.505 ± 2.067
Social influence	135	68.9%	61	31.1%	3.464 ± 2.057
Lack of energy	71	36.2%	125	63.8%	5.327 ± 2.204
Lack of willpower	92	46.9%	104	53.1%	4.434 ± 2.499
Fear of injury	179	91.3%	17	8.7%	1.306 ± 1.727
Lack of skills	164	83.7%	32	16.3%	2.087 ± 2.197
Lack of resources	59	30.1%	137	69.9%	5.556 ± 2.260
Total	130	66.3%	66	33.7%	27.678 ± 9.556

We noted a significantly higher perceived barrier of lack of time by male respondents than female respondents (p=0.048). Furthermore, female respondents showed a significant association with fear of injury (p=0.027) as a barrier to physical activity. We noted that physicians' income was associated with a lack of time (p=0.050) and resources (p=0.021). However, non-Saudi physicians had a significantly greater lack of resources for physical activity than Saudi physicians (p=0.029). Moreover, physician specialty was significantly associated with a lack of skills (p=0.041) and resources (p=0.021) (Tables 4, 5).

**Table 4:** Relationship between demographic characteristics and lack of time, social influence, lack of energy, and lack of willpower as barriers to physical activity among physicians in PHCs of Makkah city (n=196). Statistical tests: ANOVA, t-test *Statistically significant. ANOVA: analysis of variance; PHC: primary healthcare center; SD: standard deviation; SR: Saudi riyal

Characteristics		Lack of time (mean ± SD)	p-Value	Social influence (mean ± SD)	p-Value	Lack of energy (mean ± SD)	p-Value	Lack of willpower (mean ± SD)	p-Value
Gender	Male	5.72 ± 1.92	0.048*	3.54 ± 2.02	0.492	5.45 ± 2.24	0.285	4.40 ± 2.58	0.783
	Female	5.11 ± 2.27		3.33 ± 2.13		5.10 ± 2.13		4.50 ± 2.37	
Nationality	Saudi	5.55 ± 2.09	0.419	3.49 ± 2.08	0.624	5.30 ± 2.27	0.633	4.42 ± 2.52	0.827
	Non-Saudi	5.15 ± 1.90		3.25 ± 1.89		5.55 ± 1.54		4.55 ± 2.35	
Marital status	Single	5.48 ± 2.16	0.952	3.57 ± 2.16	0.914	5.85 ± 1.78	0.177	4.52 ± 2.83	0.875
	Married	5.52 ± 2.05		3.44 ± 2.02		5.15 ± 2.27		4.43 ± 2.37	
	Divorced and widowed	5.29 ± 2.06		3.29 ± 2.50		5.43 ± 2.99		4.00 ± 3.21	
Specialty	General physician	5.57 ± 2.07	0.435	3.70 ± 2.27	0.763	5.72 ± 2.17	0.190	4.38 ± 2.58	0.473
	Family medicine resident	5.23 ± 2.17		3.23 ± 2.02		4.93 ± 2.10		3.89 ± 2.67	
	Family medicine specialist	5.47 ± 2.11		3.29 ± 1.83		5.61 ± 2.17		4.82 ± 2.52	
	Family medicine consultant	5.39 ± 2.11		3.58 ± 2.12		4.76 ± 2.41		4.55 ± 2.12	
	Others	6.35 ± 1.50		3.65 ± 2.09		5.41 ± 2.12		4.71 ± 2.42	
Work experience in years	1-4	5.90 ± 1.99	0.424	3.80 ± 2.04	0.635	5.63 ± 2.02	0.214	4.71 ± 2.69	0.751
	4-7	5.24 ± 2.35		3.39 ± 1.95		5.26 ± 2.30		4.24 ± 2.52	
	7-10	5.38 ± 2.05		3.33 ± 2.20		5.68 ± 2.26		4.55 ± 2.30	
	>10	5.51 ± 1.84		3.34 ± 2.09		4.85 ± 2.20		4.28 ± 2.48	
Monthly income in SR	<10,000	4.00 ± 1.87	0.050*	3.33 ± 2.35	0.421	5.33 ± 1.80	0.264	4.44 ± 2.19	0.153
	10,000-14,999	5.56 ± 1.85		3.49 ± 1.78		5.15 ± 1.94		3.82 ± 2.57	
	15,000-20,000	5.92 ± 2.12		3.80 ± 2.26		5.79 ± 2.11		4.97 ± 2.49	
	>20,000	5.34 ± 2.08		3.23 ± 1.99		5.08 ± 2.39		4.33 ± 2.47	
Other business	Yes	5.67 ± 2.35	0.781	3.08 ± 2.50	0.509	5.67 ± 1.87	0.582	4.17 ± 2.72	0.704
	No	5.49 ± 2.05		3.49 ± 2.03		5.30 ± 2.23		4.45 ± 2.49	

**Table 5:** Relationship between demographic characteristics and fear of injury, lack of skills, and lack of resources as barriers to physical activity among physicians in PHCs of Makkah city (n=196). Statistical tests: ANOVA, t-test *Statistically significant. ANOVA: analysis of variance; PHC: primary healthcare center; SD: standard deviation; SR: Saudi riyal

Characteristics		Fear of injury (mean ± SD)	p-Value	Lack of skills (mean ± SD)	p-Value	Lack of resources (mean ± SD)	p-Value
Gender	Male	1.10 ± 1.64	0.027*	2.21 ± 2.14	0.308	5.67 ± 2.27	0.360
	Female	1.67 ± 1.83		1.87 ± 2.29		5.36 ± 2.25	
Nationality	Saudi	1.30 ± 1.76	0.905	2.08 ± 2.23	0.892	5.44 ± 2.19	0.029*
	Non-Saudi	1.35 ± 1.46		2.15 ± 1.98		6.60 ± 2.66	
Marital status	Single	1.26 ± 1.60	0.978	2.39 ± 2.43	0.493	5.61 ± 2.22	0.706
	Married	1.32 ± 1.75		2.01 ± 2.14		5.57 ± 2.30	
	Divorced and widowed	1.29 ± 2.21		1.57 ± 1.90		4.86 ± 1.77	
Specialty	General physician	1.75 ± 2.10	0.145	2.81 ± 2.26	0.041	6.36 ± 2.16	0.044
	Family medicine resident	0.84 ± 1.18		1.57 ± 2.04		5.45 ± 2.50	
	Family medicine specialist	1.27 ± 1.54		2.16 ± 2.21		5.16 ± 2.11	
	Family medicine consultant	1.27 ± 1.79		1.73 ± 2.00		5.24 ± 2.14	
	Others	1.28 ± 1.80		1.70 ± 2.09		5.18 ± 2.13	
Work experience in years	1-4	1.43 ± 1.73	0.921	2.37 ± 2.23	0.734	5.57 ± 2.39	0.527
	4-7	1.30 ± 1.79		2.09 ± 2.50		5.19 ± 2.10	
	7-10	1.33 ± 1.75		1.88 ± 2.10		5.78 ± 2.39	
	>10	1.19 ± 1.69		1.98 ± 1.92		5.75 ± 2.21	
Monthly income in SR	<10,000	1.11 ± 1.62	0.943	2.00 ± 1.94	0.248	5.33 ± 2.96	0.021*
	10,000-14,999	1.28 ± 1.56		2.21 ± 1.99		6.38 ± 2.34	
	15,000-20,000	1.41 ± 1.95		2.49 ± 2.49		5.74 ± 2.22	
	>20,000	1.26 ± 1.67		1.76 ± 2.07		5.08 ± 2.09	
Other business	Yes	0.83 ± 0.94	0.329	1.42 ± 1.38	0.277	4.92 ± 2.19	0.313
	No	1.34 ± 1.76		2.13 ± 2.24		5.60 ± 2.26	

We found no significant relationship between sociodemographic characteristics and physical activity barriers among physicians (Table 6). However, we noted a significant negative correlation between owning a gym membership and high-score barriers (p<0.001), vigorous activities and perceived barriers (p<0.001), and between moderate physical activity and perceived barriers (p<0.001).

**Table 6:** Association between the barrier to physical activities and sociodemographic data among physicians in PHCs of Makkah city. *Statistically significant. ANOVA: analysis of variance; PHC: primary healthcare center; SD: standard deviation; SR: Saudi riyal

Items	Classification	N	Barriers (mean ± SD)	ANOVA or t-test (p-Value)
Gender	Male	70	28.087 ± 9.212	0.423
	Female	126	26.943 ± 10.172	
Nationality	Saudi	176	27.574 ± 9.693	0.650
	Non-Saudi	20	28.600 ± 8.419	
Marital status	Single	46	28.674 ± 9.895	0.648
	Married	143	27.455 ± 9.372	
	Divorced and widowed	7	25.712 ± 11.883	
Specialty	General physician	53	30.28 ± 10.38	0.107
	Family medicine resident	44	25.14 ± 9.74	
	Family medicine specialist	49	27.78 ± 9.50	
	Family medicine consultant	33	26.52 ± 7.65	
	Others	17	28.12 ± 8.74	
Work experience in years	1-4	49	29.408 ± 9.906	0.472
	4-7	54	26.704 ± 10.597	
	7-10	40	27.900 ± 9.248	
	>10	53	26.906 ± 8.296	
Monthly income in SR	<10,000	9	25.556 ± 8.618	0.076
	10,000-14,999	39	27.897 ± 8.614	
	15,000-20,000	61	30.115 ± 10.437	
	>20,000	87	26.092 ± 9.171	
Other business	Yes	12	25.750 ± 10.226	0.472
	No	184	27.804 ± 9.527	
Chronic disease	Yes	29	27.828 ± 10.007	0.928
	No	167	27.653 ± 9.506	
Smoking	Regularly	18	27.056 ± 7.619	0.825
	Occasionally	36	26.556 ± 9.793	
	Ex-smoker	14	28.929 ± 9.294	
	Never smoked	128	27.945 ± 9.824	
Gym membership	Yes	60	23.983 ± 8.715	<0.001*
	No	136	29.309 ± 9.485	
Vigorous physical activity	Yes	55	22.564 ± 8.819	<0.001*
	No	141	29.674 ± 9.105	
Moderate physical activity	Yes	60	24.233 ± 9.201	<0.001*
	No	136	29.199 ± 9.344	
Walking	Yes	137	27.204 ± 9.702	0.291
	No	59	28.780 ± 9.193	

Table 7 shows a significant negative correlation between BMI and lack of resources (p=0.041, r= -0.146). Age did not have any significant correlation with barriers to physical activity. Duration of sitting was significantly correlated with lack of resources (p=0.018, r=0.169) and fear of injuries (p=0.009, r= -0.185).

**Table 7:** Association between the sociodemographic data and barriers to physical activity among physicians in PHCs of Makkah city. *Statistically significant. BMI: body mass index; PHC: primary healthcare center

Items		BMI	Age	Duration of sitting
Lack of time	χ2	-0.103	-0.066	0.068
	p-value	0.149	0.361	0.340
Social influence	χ2	0.015	-0.062	0.085
	p-value	0.838	0.391	0.234
Lack of energy	χ2	-0.029	-0.089	0.054
	p-value	0.690	0.212	0.454
Lack of willpower	χ2	0.043	-0.001	-0.028
	p-value	0.546	0.985	0.695
Fear of injury	χ2	0.116	0.040	-0.185
	p-value	0.106	0.581	0.009*
Lack of skills	χ2	0.009	-0.027	0.048
	p-value	0.904	0.708	0.506
Lack of resources	χ2	-0.146	0.130	0.169
	p-value	0.041*	0.070	0.018*
Total	χ2	-0.026	-0.017	0.056
	p-value	0.716	0.816	0.438

## Discussion

We conducted this study to assess physicians' physical activity levels and investigate the perceived barriers to exercise. A low percentage of physicians engaged in physical activity cited lack of time, resources, energy, and willpower as perceived barriers to physical activity. Our findings are comparable to a cross-sectional study done among doctors working in King Abdulaziz Hospital in Jeddah that used the IPAQ and the Exercise Benefits/Barriers Scale. In that study, 36.4% and 41.6% of physicians engaged in vigorous and moderate activity, respectively [2]. Another study done in Al-Jouf region among physicians working in PHCs found that 44.3% of respondents did moderate and 20.8% were doing vigorous physical exercise [8]. A 2019 study on Portuguese adults reported that the prevalence of moderate to vigorous physical activity in that population was 30% and 27%, respectively [20]. In 2012, a study conducted in Poland among doctors showed only 18.5% reported engaging in vigorous activity, and 77.4% had regular moderate activity [21]. Therefore, the prevalence of physical activity among physicians in Makkah PHCs is comparably low.

This low prevalence might be related to sedentary lifestyles or perceived barriers. Major barriers reported by physicians are a lack of time, motivation, and a physical activity partner [15,22]. A United Kingdom study showed that lack of time was the most significant barrier to physical activity among medical and nursing students [23]. A study among physicians in Riyadh concluded that the main barriers to exercise were limited exercise facilities at home and harsh weather [24]. Another study in the southwestern region of Saudi Arabia among healthcare students reported major barriers were lack of time and lack of accessible and suitable sports facilities [4]. 

We found a significant association between prolonged sitting, lack of resources, and fear of injuries. High BMI was significantly correlated with a lack of resources, too. Age had no significant correlation with barriers to physical activity. However, in some global studies, age was an important variable in predicting physical activity patterns as younger medical students were much more physically active than older ones [25]. A 2001 study in Riyadh showed a significant association between age, gender, and lack of time [7]. In Australia, a study published in 2013 showed a significant association between sitting hours and lack of time for exercise [26]. In a study conducted recently among physicians and nurses in Egypt, there was a significant correlation between specialty and lack of time [23].

In Saudi Arabia, there are significant moves toward healthy lifestyles; the MOH has launched several healthy activity initiatives that include a walking challenge and a program called "know your numbers" which aims to raise awareness about the importance of knowing and monitoring four vital signs that affect an individual’s health: blood glucose, blood pressure, waist circumference, and BMI [27,28]. There are initiatives to build more gyms and walking areas in many Saudi regions. Few gyms operate for 24 hours, which can be helpful for physicians to have time to exercise outside of their work schedules. Likewise, physicians should model healthy lifestyle choices, including a good diet, physical activity levels, and adequate sleep. 

While our study used a validated questionnaire approved by the CDC, it was not without limitations. As with any cross-sectional study, the reported values are a snapshot and do not represent the full picture of this population. The study did not include the effect of being a physically active physician on counseling patients to change their behaviors. Also, the exercise period may be inaccurate because the time units changed across different questions.

## Conclusions

This study aimed to estimate the physical activity levels of physicians working in the PHCs of Makkah and any perceived barriers to physical exercise. Even though Saudi Arabia is trying to increase awareness of healthy lifestyle choices among all residents, most physicians were not physically active, according to our study. The main barriers to physical activity were lack of time and resources. Gender, nationality, specialty, and income had a significant association with barriers to physical activity. Long periods of sitting significantly correlated with fear of injuries and lack of resources for physical activity. Physicians are health role models for the population and the representative face of the healthcare system. Physicians should be encouraged and motivated to be physically active to model healthy choices for patients and the general population.
